# Postmortem evidence of decreased brain pH in major depressive disorder: a systematic review and meta-analysis

**DOI:** 10.1038/s41398-024-03173-7

**Published:** 2024-11-04

**Authors:** Hideo Hagihara, Tsuyoshi Miyakawa

**Affiliations:** Division of Systems Medical Science, Center for Medical Science, Fujita Health University, Toyoake, Japan

**Keywords:** Molecular neuroscience, Depression

## Abstract

**Introduction:**

Major depressive disorder (MDD) is a prevalent and debilitating mental disorder that shares symptoms, genetics, and molecular changes in the brain with other psychiatric disorders, such as schizophrenia and bipolar disorder. Decreased brain pH, associated with increased lactate levels due to altered energy metabolism and neuronal hyperexcitation, has been consistently observed in schizophrenia and bipolar disorder. We recently demonstrated similar brain alterations in various animal models of neuropsychiatric disorders, including MDD. However, our understanding of brain pH alterations in human patients with MDD remains limited.

**Methods:**

We conducted meta-analyses to assess postmortem brain pH in patients with MDD compared to control subjects, examining its relationships with recurrence of depressive episodes and illness duration, utilizing publicly available demographic data. Studies reporting individual raw pH data were identified through searches in the Stanley Medical Research Institute database, NCBI GEO database, PubMed, and Google Scholar. The data were analyzed using the random effects model, ANOVA, and ANCOVA.

**Results:**

The random effects model, using 39 curated datasets (790 patients and 957 controls), indicated a significant decrease in brain pH in patients with MDD (Hedges’ *g* = −0.23, *p* = 0.0056). A two-way ANCOVA revealed that the effect of diagnosis on pH remained significant when considering covariates, including postmortem interval, age at death, and sex. Patients with recurrent episodes, but not a single episode, showed significantly lower pH than controls in both females and males (256 patients and 279 controls from seven datasets). Furthermore, a significant negative correlation was observed between brain pH and illness duration (115 patients from five datasets). Female preponderance of decreased pH was also found, possibly due to a longer illness duration and a higher tendency of recurrent episodes in females.

**Conclusion:**

This study suggests a decrease in brain pH in patients with MDD, potentially associated with recurrent episodes and longer illness duration. As suggested from previous animal model studies, altered brain energy metabolism, leading to decreased pH, may serve as a potential transdiagnostic endophenotype for MDD and other neuropsychiatric disorders.

## Introduction

Accumulating evidence suggests that decreased brain pH is a common feature of several psychiatric disorders, such as schizophrenia and bipolar disorder [1, 2]. Recent meta-analyses of magnetic resonance spectrometry (MRS) [3] and postmortem studies [4] have reported statistically significant and marginal decreases in brain pH in patients with these disorders compared with healthy controls. Decreased brain pH has frequently been associated with increased lactate levels under these psychiatric conditions [5–7]. Increased lactate levels are considered indicative of metabolic changes resulting from mitochondrial dysfunction and/or increased glycolysis due to neuronal hyperexcitation. It has been reported that tissue pH influences gene expression in the brain [8], and genes sensitive to pH are linked to various biological processes relevant to these disorders, such as energy production, the immune system, and synaptic signaling [9, 10]. Considering the strict regulation of pH in the brain, changes in pH could be a potential clue to understanding the pathophysiology of these disorders [11].

Major depressive disorder (MDD) is a common mood disorder that partially shares clinical and neurobiological features with schizophrenia and bipolar disorder, such as cognitive dysfunction [12–14], functional dysconnectivity in certain brain networks [15, 16], and genetic risks [17]. Our recent large-scale animal studies demonstrated that decreased brain pH and increased lactate levels are common hallmarks among many animal models of depressive disorders and other neuropsychiatric disorders, including schizophrenia, bipolar disorder, autism spectrum disorder, and Alzheimer’s disease [4, 18]. Animal models of depressive disorders included serotonin transporter knockout (SERT KO) mice [19, 20], mice exposed to social defeat stress [21, 22], and mice induced to develop diabetes mellitus or colitis, which have a high comorbidity risk for depression [23, 24]. An increase in brain lactate levels has been suggested in patients with MDD compared to control subjects by MRS studies [25, 26]. However, little is known about brain pH in MDD. The pH of tissue of individual subjects is frequently reported in the demographic data of samples in human studies using postmortem brains, as it is considered an indicator of the quality of preserved tissue. In this study, we conducted meta-analyses utilizing publicly available data to investigate postmortem brain pH in MDD.

## Methods

### Identification and selection of eligible postmortem brain pH datasets

**Fig. 1:**
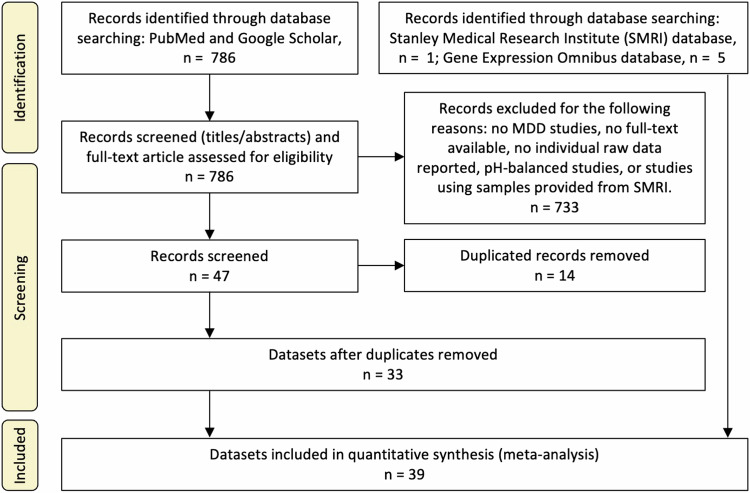
Workflow of the selection of postmortem brain pH datasets. The studies that reported matching brain tissue pH between patient and control groups and/or excluding samples with a pH below a certain threshold were considered as pH-balanced studies.

Datasets were selected based on the availability of raw pH data of individual subjects. We obtained pH data of patients with MDD and control subjects from the Stanley Medical Research Institute (SMRI) database (https://www.stanleygenomics.org) [27]. Furthermore, we searched the National Center for Biotechnology Information (NCBI) Gene Expression Omnibus database (GEO), PubMed, and Google Scholar for studies reporting individual pH data with the key words ‘brain pH’, ‘postmortem’ and ‘major depression’. Data searches followed the Preferred Reporting Items for Systematic Reviews and Meta-Analyses guidelines [28] and were carried out by an author (HH) from September 15 to 27, 2023. Figure 1 presents the workflow of the selection of postmortem brain pH datasets. Articles were screened first based on their titles, followed by a subsequent evaluation of their abstracts to determine eligibility for full-text review. The database search retrieved 792 records. For records identified in PubMed and Google Scholar, inclusion criteria consisted of studies involving human MDD versus control comparisons (CON) using postmortem brain samples and the availability of full text and individual raw data. Changes in postmortem brain pH have been considered an artifact due to pre- and postmortem confounding factors [5, 29] and, hence, considerable efforts have been made to match tissue pH between patient and control groups within studies. Such a sample selection based on pH may influence the meta-analysis, potentially biasing the results towards showing no difference between MDD and controls. Therefore, in this study, we excluded studies that reported matching brain tissue pH between the patient and control groups and/or excluding samples with a pH below a certain threshold (Fig. 1). To avoid duplication, studies that used brain samples provided by SMRI were also excluded from the studies retrieved through NCBI PubMed and Google Scholar searches. To minimize the inclusion of overlapping samples across different studies, we attempted to identify and remove duplicated samples from studies that used partially overlapping samples from the same source, and then combined the remaining data into a single dataset. If conditions other than CON and MDD were present within the study, they were excluded from the current analysis. Altogether, 39 datasets were included in the present study (Table 1). We also obtained the accompanying demographic information from the datasets, including postmortem interval (PMI), age at death, sex, duration of illness, RNA integrity number (RIN), manner of death (suicide or non-suicide), recurrence (single or recurrent episodes), and disease state at the time of death (remitted or depressed) (Table S1).

**Table 1:** Summary of datasets used in this study.

Dataset and source/reference	Diagnosis group	Number of samples (Female/Male)	Brain pH^a^	Brain region for pH measurement^b^	PMI (hours)^a^	Age (years)^a^	Duration of illness (years)^a^
Kaul 2020 [56]	CON MDD	8 (3/5) 5 (3/2)	6.77 ± 0.037 6.44 ± 0.113	N/A	32.2 ± 3.6 33.8 ± 3.2	55.1 ± 5.5 57.4 ± 7.2	N/A
GSE35977, NCBI GEO	CON MDD	49 (16/34) 14 (6/8)	6.50 ± 0.044 6.19 ± 0.058	N/A	27.2 ± 1.7 27.6 ± 3.0	45.4 ± 1.3 46.1 ± 2.6	N/A
Xue 2022 [57]	CON MDD	22 (10/12) 6 (3/3)	6.64 ± 0.076 6.38 ± 0.060	N/A	15.7 ± 1.4 16.3 ± 2.6	47.3 2.1 50.5 ± 3.0	N/A
Rajkowaska 2005 [58]	CON MDD	19 (7/12) 23 (10/13)	6.77 ± 0.031 6.57 ± 0.045	N/A	20.7 ± 1.2 21.6 ± 1.1	58.4 ± 4.2 63.7 ± 3.7	20.7 ± 4.9
Vinod 2010 [59]	CON MDD	22 (0/22) 3 (0/3)	6.63 ± 0.042 6.52 ± 0.052	Cerebellum	13.5 ± 1.0 10.7 ± 2.2	39.9 ± 3.4 33.0 ± 3.5	N/A
Xiang 2008 [60]	CON MDD	11 (N/A) 11 (N/A)	6.66 ± 0.079 6.51 ± 0.071	N/A	17.4 ± 1.7 20.5 ± 1.2	N/A N/A	N/A
Gray 2015 [61]	CON MDD	32 (13/19) 53 (27/26)	6.54 ± 0.050 6.36 ± 0.041	DLPFC	29.1 ± 2.3 36.2 ± 3.4	39.2 ± 2.6 43.7 ± 2.1	N/A
Douillard-Guilloux 2013, 2016 [62, 63]	CON MDD	22 (22/0) 22 (22/0)	6.71 ± 0.050 6.56 ± 0.062	N/A	16.5 ± 1.3 17.1 ± 1.1	47.1 ± 3.1 45.4 ± 2.9	N/A
Pandey 2018, 2019; Singh 2020 [64–66]	CON MDD	27 (6/21) 36 (15/11)	7.01 ± 0.027 6.91 ± 0.044	N/A	17.3 ± 1.3 18.6 ± 1.2	43.8 ± 3.1 42.5 ± 2.8	N/A
Dienel 2023 [67]	CON MDD	43 (18/25) 42 (18/24)	6.65 ± 0.038 6.54 ± 0.032	The mean of prefrontal and cerebellar or occipital pH values	19.3 ± 0.9 19.4 ± 0.9	47.6 ± 2.1 45.6 ± 1.9	12.0 ± 1.6
Shelton 2009, 2011 [68, 69]	CON MDD	20 (3/17) 20 (3/17)	6.72 ± 0.061 6.57 ± 0.077	N/A	17.9 ± 1.2 18.2 ± 1.4	46.9 ± 3.0 45.5 ± 3.2	N/A
Dwivedi 2003 [70]	CON MDD	24 (4/20) 11 (6/5)	6.10 ± 0.072 5.93 ± 0.13	N/A	18.1 ± 1.6 17.8 ± 2.0	42.4 ± 2.8 36.2 ± 3.3	N/A
Chung 2018 [71]	CON MDD	40 (18/22) 40 (18/22)	6.64 ± 0.040 6.54 ± 0.036	N/A	19.6 ± 0.9 19.2 ± 0.9	46.8 ± 2.0 45.5 ± 1.8	N/A
Karolewicz 2004 [72]	CON MDD	12 (2/10) 12 (3/9)	6.69 ± 0.066 6.58 ± 0.082	N/A	18.5 ± 1.2 25.5 ± 2.2	46.5 ± 3.8 48.5 ± 4.4	N/A
SMRI_C, SMRI	CON MDD	15 (6/9) 15 (6/9)	6.27 ± 0.062 6.18 ± 0.055	Occipital cortex	23.7 ± 2.6 27.5 ± 2.8	48.1 ± 2.8 46.5 ± 2.4	N/A
Underwood 2008 [73]	CON MDD	19 (3/16) 2 (0/2)	6.68 ± 0.054 6.55 ± 0.313	N/A	17.3 ± 1.0 12.0 ± 3.0	44.0 ± 3.7 31.5 ± 11.5	N/A
GSE101521, NCBI GEO	CON MDD	29 (6/23) 30 (11/19)	6.49 ± 0.062 6.41 ± 0.055	N/A	13.2 ± 0.9 15.8 ± 1.2	43.5 ± 3.9 53.7 ± 3.6	N/A
Seney 2018 [74]	CON MDD	50 (24/26) 51 (24/27)	6.74 ± 0.028 6.68 ± 0.038	N/A	16.9 ± 0.9 16.3 ± 0.8	49.9 ± 1.8 49.7 ± 1.8	N/A
Sharma 2022 [75]	CON MDD	24 (4/20) 24 (10/14)	7.02 ± 0.030 6.96 ± 0.052	N/A	16.5 ± 1.3 18.9 ± 1.2	42.1 ± 3.1 39.0 ± 3.1	N/A
Martín-Hernández 2018 [76]	CON MDD	15 (3/12) 15 (3/12)	6.53 ± 0.114 6.45 ± 0.114	DLPFC	19.9 ± 3.0 19.3 ± 1.9	40.5 ± 2.7 41.7 ± 2.6	N/A
Feyissa 2009, 2010 [77, 78]	CON MDD	16 (4/12) 17 (5/12)	6.67 ± 0.071 6.61 ± 0.068	N/A	19.9 ± 1.5 23.3 ± 1.3	49.9 ± 4.1 49.9 ± 3.6	N/A
Pandey 2013 [79]	CON MDD	29 (8/21) 8 (2/6)	6.17 ± 0.073 6.08 ± 0.164	N/A	17.4 ± 1.4 17.9 ± 2.0	16.2 ± 0.3 15.8 ± 0.8	N/A
Roy 2017 [80]	CON MDD	11 (2/9) 9 (3/6)	6.82 ± 0.043 6.79 ± 0.058	N/A	4.0 ± 0.5 5.8 ± 1.4	66.9 ± 4.2 45.2 ± 4.9	N/A
Di Narzo 2014 [81]	CON MDD	31 (21/10) 34 (24/10)	6.62 ± 0.043 6.57 ± 0.045	ACC	17.8 ± 1.0 17.2 ± 0.8	49.3 ± 2.5 49.1 ± 2.2	N/A
Arion 2017 [82]	CON MDD	19 (9/10) 19 (9/10)	6.61 ± 0.052 6.56 ± 0.054	N/A	19.3 ± 1.2 20.1 ± 1.4	47.5 ± 2.4 45.2 ± 2.3	N/A
Tripp 2011, 2012; Sibille 2011; Dowling 2023 [83–86]	CON MDD	76 (32/44) 73 (33/40)	6.66 ± 0.028 6.61 ± 0.029	N/A	17.7 ± 0.7 17.3 ± 0.6	48.9 ± 1.5 48.1 ± 1.4	10.1 ± 2.1
Wang 2017 [87]	CON MDD	11 (3/8) 11 (2/9)	6.86 ± 0.041 6.84 ± 0.061	Cerebellum	17.5 ± 1.6 19.2 ± 2.5	39.5 ± 4.4 48.4 ± 3.9	N/A
GSE53987, NCBI GEO	CON MDD	19 (9/10) 18 (8/10)	6.59 ± 0.050 6.58 ± 0.055	N/A	19.5 ± 1.2 19.9 ± 1.4	48.1 ± 2.4 45.1 ± 2.5	N/A
Bach 2014 [88]	CON MDD	10 (2/8) 3 (0/3)	6.60 ± 0.119 6.58 ± 0.117	Cerebellum	12.7 ± 1.6 9.7 ± 1.5	39.5 ± 6.7 41.0 ± 13.6	N/A
Szewczyk 2009, 2010 [89, 90]	CON MDD	28 (15/13) 27 (14/13)	6.64 ± 0.045 6.63 ± 0.042	N/A	18.6 ± 1.4 19.9 ± 1.5	54.3 ± 3.1 54.6 ± 3.2	16.3 ± 2.8
Chandley 2022 [91]	CON MDD	22 (2/20) 21 (2/19)	6.55 ± 0.066 6.59 ± 0.047	Occipital Cortex, Locus Coeruleus	21.4 ± 1.3 21.1 ± 1.6	N/A N/A	N/A
GSE92538, NCBI GEO	CON MDD	72 (17/55) 40 (8/32)	6.81 ± 0.033 6.86 ± 0.045	Cerebellum	22.7 ± 0.9 26.2 ± 1.2	57.4 ± 1.5 48.5 ± 2.5	N/A
Boldrini 2009 [92]	CON MDD	7 (3/4) 12 (4/8)	6.58 ± 0.115 6.67 ± 0.081	Cerebellum	15.2 ± 2.0 15.9 ± 2.0	35.9 ± 4.9 41.9 ± 4.3	N/A
Bach-Mizrachi 2006, 2008 [93, 94]	CON MDD	15 (3/12) 8 (1/7)	6.48 ± 0.110 6.57 ± 0.081	Cerebellum	14.2 ± 1.5 21.8 ± 1.3	53.3 ± 4.4 55.8 ± 5.8	N/A
Dean 2016 [95]	CON MDD	20 (9/11) 9 (5/4)	6.29 ± 0.045 6.40 ± 0.117	N/A	42.1 ± 3.8 48.5 ± 5.7	50.4 ± 4.1 53.1 ± 5.9	16.9 ± 3.3
Zhurov 2012 [96]	CON MDD	9 (N/A) 10 (N/A)	6.51 ± 0.092 6.63 ± 0.066	N/A	3.7 ± 0.7 5.3 ± 1.3	59.4 ± 5.1 52.5 ± 2.8	N/A
Evans 2004 [97]	CON MDD	13 (2/11) 13 (3/10)	6.92 ± 0.037 6.99 ± 0.047	N/A	22.2 ± 2.0 25.4 ± 1.8	53.2 ± 4.4 50.8 ± 4.4	N/A
Mamdani 2014 [98]	CON MDD	10 (3/7) 10 (7/3)	6.15 ± 0.059 6.41 ± 0.123	N/A	20.5 ± 2.6 24.8 ± 2.4	48.0 ± 4.1 47.3 ± 3.6	N/A
GSE208338, NCBI GEO	CON MDD	62 (13/49) 24 (12/12)	6.37 ± 0.025 6.54 ± 0.036	N/A	41.9 ± 1.9 39.0 ± 2.9	49.3 ± 2.0 54.4 ± 3.7	N/A

^a^mean ± sem.

^b^Studies that did not clearly specify which brain regions’ pH was analyzed are marked as N/A.

*ACC* Anterior cingulate cortex, *CON* control, *DLPFC* Dorsolateral prefrontal cortex, *MDD* major depressive disorder, *N/A* Not available, *NCBI GEO* National Center for Biotechnology Information Gene Expression Omnibus database, *PMI* postmortem interval, *SMRI_C* Stanley Medical Research Institute database Collection C.

### Data analysis

The standardized mean difference (SMD) of pH between patients and control subjects and the correlation coefficient between pH and duration of illness in patients were calculated within each dataset. The random effects model was applied using the function metagen in the R package meta (version 6.5-0). Heterogeneity was measured by calculating *I*^2^. The results were visualized by means of forest plots using the function forest of R. The sensitivity analysis was conducted to assess the relative influence of each dataset on the pooled estimate by omitting one dataset at a time using the random effects model. Publication bias was assessed by a funnel plot and the Egger’s test using the function funnel and metabias of R, respectively. Analysis of covariance (ANCOVA) was performed using the GLM procedure in SAS (version 3.81; SAS Institute Inc., Cary, NC, USA). Unpaired t-test and analysis of variance (ANOVA) were performed using GraphPad Prism 8 (version 8.4.2; GraphPad Software, San Diego, CA, USA). Subgroup analyses of brain pH levels, considering the recurrence of episodes, illness duration, manner of death, state at the time of death, regional difference, and RIN, were conducted using the respective datasets that reported this information. The raw data analyzed in this study are provided in Table S1.

## Results

### Search results

Following screening the 786 records retrieved in PubMed and Google Scholar searches, 47 studies were identified (Fig. 1). After removing duplicates (14 studies) and including studies identified in the SMRI (one study) and NCBI GEO databases (five studies), a total of 39 datasets were processed for the meta-analysis. Funnel plot and Egger’s test showed no evidence of publication bias for the included studies (*t* = 0.39, *p* = 0.70; Fig. S1). Table 1 presents a summary of the datasets, which contains information on brain pH, brain region used for pH measurement, PMI, age at death, sex, and duration of illness. The source and references of the datasets used are also included in Table 1.

### Decreased postmortem brain pH in patients with MDD

**Fig. 2:**
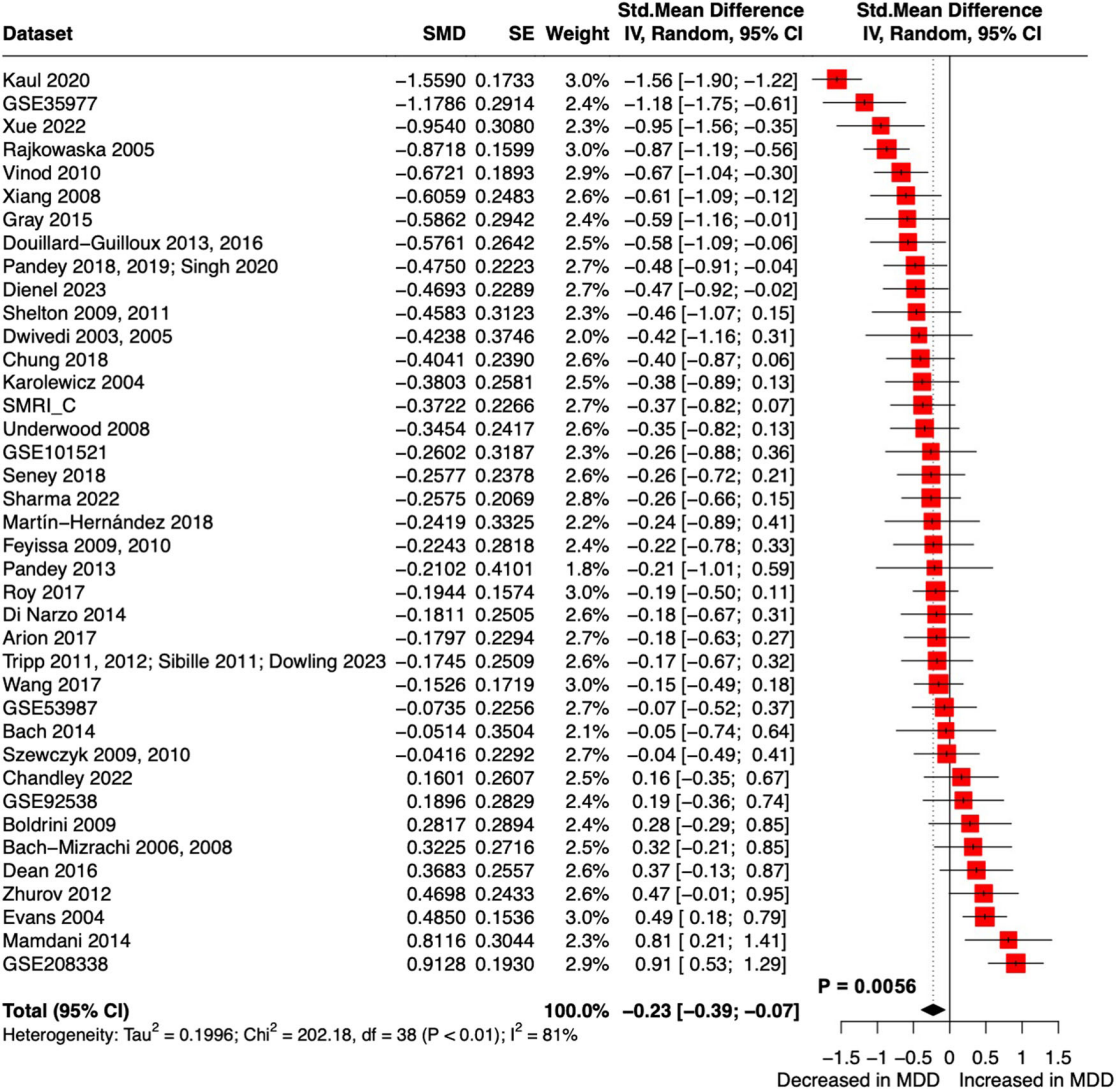
Decreased pH in the postmortem brains of patients with MDD. Forest plot of meta-analysis comparing postmortem brain pH between patients with MDD and control individuals. 95% CI, 95% confidence interval; SE, standard error; SMD, standardized mean difference.

**Fig. 3:**
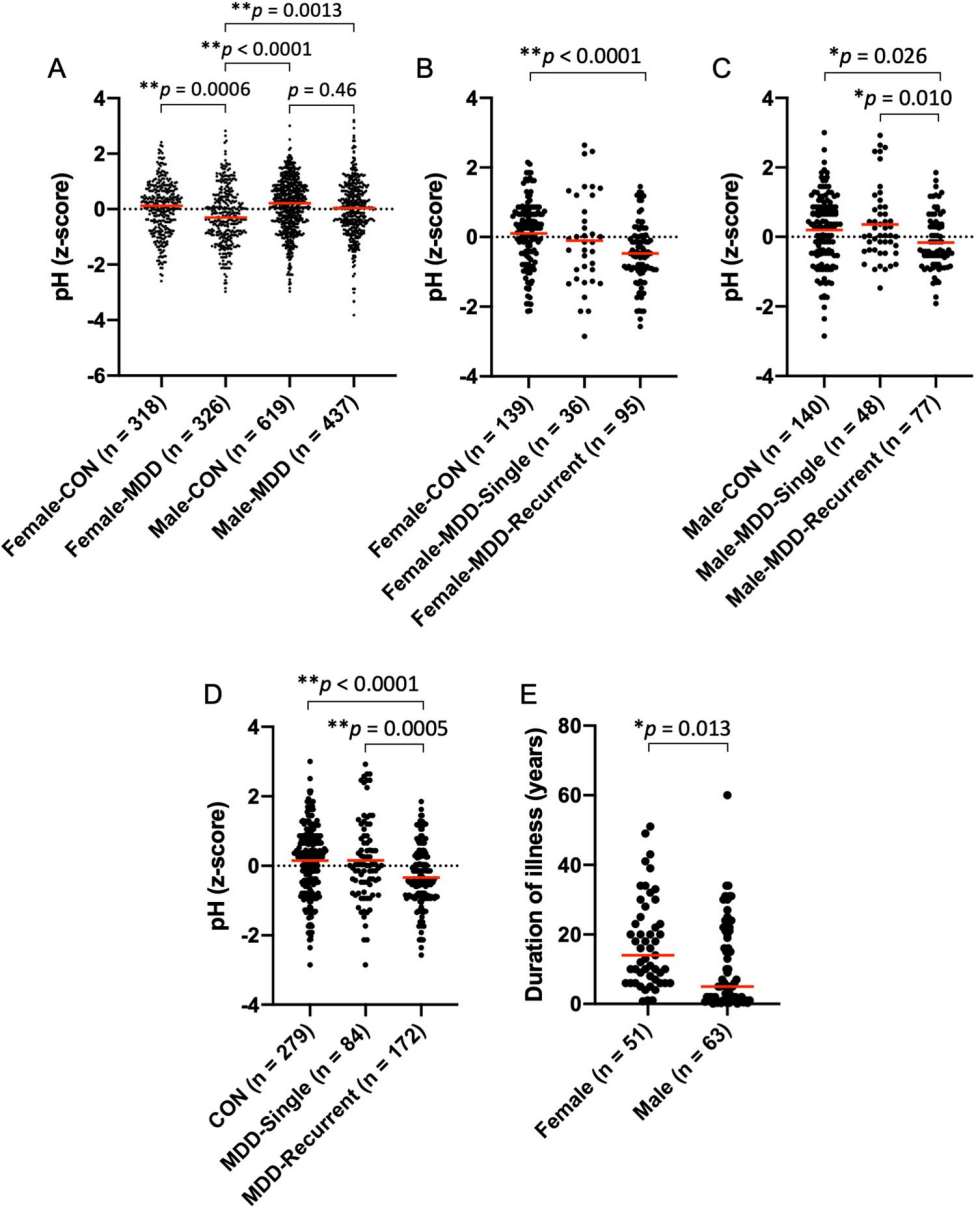
Decreased brain pH in female patients associated with recurrent episodes and illness duration in MDD. **A** Scatter plot of z-score-transformed pH values based on sex and diagnosis. The z-scores were calculated within each dataset. Two-way ANOVA: effect of sex: *p* = 0.0007; effect of diagnosis: *p* < 0.0001; interaction: *p* = 0.043. Red bars indicate mean values for each group. The p-values from Sidak’s multiple comparison test following the two-way ANOVA are shown. **B–D** Effect of recurrence of depression episodes on brain pH levels by sex. Scatter plot of z-score-transformed pH values of control subjects, patients with MDD with a single episode, and those with recurrent episodes of depression in the female datasets (**B**), male datasets (C), and combined female and male datasets (**D**). A total of 535 samples from seven datasets were analyzed. The z-scores were calculated within each dataset. The p-values from Tukey’s multiple comparison test following the one-way ANOVA are shown. **E** Scatter plot of the duration of illness by sex. A total of 114 samples from five datasets were analyzed. The p-value from the unpaired t-test is shown. CON, control subjects; MDD, major depressive disorder.

Of the 39 datasets obtained, five showed a significant decrease in brain pH in patients with MDD when compared to the corresponding controls within those datasets (Dienel 2023, *p* = 0.032; Gray 2015, *p* = 0.010; GSE35977, *p* = 0.00062; Kaul 2020, *p* = 0.0074; Rajkowaska 2005, *p* = 0.041; two-tailed unpaired t-test), and one showed an increase in pH (GSE208338, *p* = 0.016). The others showed no significant difference in pH between patients and controls. The meta-analysis using the random effects model revealed a significant decrease in brain pH in patients with MDD when compared control subjects (Hedges’ *g* = −0.23, 95% confidence interval = [−0.39; −0.077], *p* = 0.0056) (Fig. 2). The *I*^*2*^ test showed high heterogeneity suggesting significant differences between datasets (*I*^*2*^ = 81%, *p* < 0.01). Similar results were obtained in a sensitivity analysis with each dataset omitted in the random effects model (Table S2), further strengthening the confidence in the observed decrease in brain pH in patients with MDD. In a two-way ANCOVA, with the diagnosis and dataset as the main factors, the effect of diagnosis (mean square (MS) = 0.83, *F* = 12.60, *p* = 0.0004) and dataset (MS = 1.63, *F* = 24.66, *p* < 0.0001) were significant. While no significant effect was observed for PMI (MS = 0.18, *F* = 2.66, *p* = 0.10) or age at death (MS = 0.20, *F* = 3.07, *p* = 0.080) in the two-way ANCOVA, the effect of sex was significant (MS = 0.93, *F* = 14.00, *p* = 0.0002). Based on the z-score-transformed data, a two-way ANOVA followed by post-hoc Sidak’s multiple comparison test revealed lower pH in female patients with MDD compared to female controls (*p* = 0.0006), but no significant difference was observed between male patients and male controls (Fig. 3A). The MDD group showed a higher proportion of females compared to the control group (χ^2^ = 13.43, *p* < 0.01). This variation in gender distribution may influence the significant effect of sex on brain pH. These results suggest that, while brain pH varied between datasets, it decreased especially in female patients with MDD compared to controls, independently of the covariates examined.

We conducted subgroup analyses to further assess the factors that may influence the potential female preponderance of decreased brain pH in MDD. Analyses considering the recurrence of depressive episodes revealed that female patients with recurrent episodes showed significantly lower brain pH compared to female controls (*p* < 0.0001, post-hoc multiple comparison test following one-way ANOVA; Fig. 3B). We found that male patients with recurrent episodes showed significantly lower brain pH compared to male patients with a single episode (*p* = 0.010) and controls (*p* = 0.026) (Fig. 3C). In the combined analysis of female and male datasets, patients with recurrent episodes showed significantly lower brain pH compared to those with a single episode (*p* = 0.0005) and controls (*p* < 0.0001) (Fig. 3D). There was no significant difference between patients with a single episode and control subjects either in females or males. These results suggest that decreased brain pH is associated with experiencing recurrent episodes, regardless of gender. We also found that female patients had a trend toward a larger number of subjects with recurrent episodes (χ^2^ = 3.46, *p* = 0.063, chi-square test) and a significantly longer duration of illness (Fig. 3E; see the following paragraph regarding the effect of illness duration on brain pH changes) compared to male patients. These factors may confound the observed female preponderance of decreased brain pH in MDD (Fig. 3A).

**Fig. 4:**
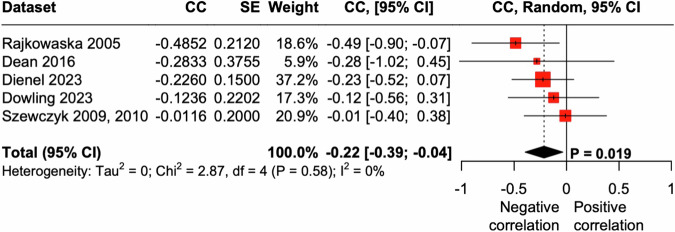
Negative correlation between brain pH and duration of illness. Forest plot of meta-analysis of correlation between brain pH and duration of illness in patients with MDD. 95% CI, 95% confidence interval; CC, correlation coefficient; SE, standard error.

Furthermore, we investigated the relationship between degree of brain pH changes and duration of illness. Information about the duration of the illness was available in five datasets (Table 1). A meta-analysis of the five datasets revealed a significant negative correlation between brain pH and the duration of illness (overall *r* = −0.22, 95% confidence interval = [−0.39; −0.04], *p* = 0.019; Fig. 4), suggesting that brain pH progressively decreases as the duration of suffering from MDD prolongs.

### Relationships between brain pH and other accompanying variables

We also conducted subgroup analyses to assess the relationship between brain pH and available accompanying variables, including the manner of death, the state at the time of death, RNA integrity, and brain region.

#### Manner of death

Subdividing the depressive patients based on the manner of death, either suicide or non-suicide, and sex, we examined the effects of these factors on pH levels. In the combined female and male datasets, non-suicide patients showed significantly lower pH compared to suicide patients and controls (*p* < 0.0001, respectively, post-hoc multiple comparison test following one-way ANOVA) (Fig. S2A). In the female datasets, non-suicide patients showed significantly lower pH compared to suicide patients (*p* = 0.0003) and controls (*p* < 0.0001) (Fig. S2B). In the male datasets, no significant difference in pH levels was observed among the suicide, non-suicide, and control groups (Fig. S2C). The decreased pH in non-suicide patients in the combined and female datasets, but not in the male datasets, may be due to a significantly higher ratio of subjects with recurrent episodes in the non-suicide group within the combined (χ^2^ = 8.44, *p* = 0.0037, chi-square test) and female datasets (χ^2^ = 10.71, *p* = 0.0011), but not in the male datasets (χ^2^ = 0.63, *p* = 0.43).

#### State at the time of death

We examined the effect of the state at the time of death, including full or partial remission, mild or moderate, and severe states, despite the small number of samples available. The results showed no significant effect of this factor on brain pH levels either in the combined, female, and male datasets (on-way ANOVA; Fig. S2D–F).

#### RNA integrity

Tissue pH has been considered a quality-control indicator of human postmortem brain tissues, based on the findings of its significant positive correlation with the RNA integrity number (RIN), where low RIN values indicate poor quality RNA [30, 31]. However, other studies have shown no significant correlation [32] and rather a negative correlation [33] between the variables, suggesting that this notion remains controversial. Our analysis demonstrated a slight but significant positive correlation between these variables (*r* = 0.16, *p* < 0.0001, Pearson’s correlation test; Fig. S2G). Notably, there was no significant effect of diagnosis or sex on RIN (Fig. S2H), suggesting that the observed decrease in brain pH in patients with MDD observed above may not be due to tissue degradation.

#### Brain region

In the analysis considering regional differences, we found a significant decrease in pH in the cerebral cortical samples (including dorsolateral prefrontal cortex, occipital cortex, and anterior cingulate cortex) of patients with MDD compared to controls (*p* = 0.029, post-hoc multiple comparison test following one-way ANOVA; Fig. S3A), while no significant difference was found in the cerebellar samples (Fig. S3D). When examining pH levels in each brain region by sex, no significant differences were found between patients with MDD and controls in any group, likely due to small sample sizes (Fig. S3B, C, E, F).

## Discussion

Our meta-analyses suggested that brain pH is decreased in patients with MDD compared to control subjects even when PMI, age, and sex were considered as potential confounding factors. We recently showed that the decrease in brain pH in MDD has been estimated through the analysis of gene expression patterns [10], which aligns with the findings of this study. Furthermore, the increased levels of brain lactate measured by MRS imaging in patients with MDD [25, 26] provide additional support for these findings.

Decreased brain pH has been implicated in several neuropsychiatric disorders, such as schizophrenia and bipolar disorder [1, 2, 34]. Our current results extend this notion to MDD, suggesting decreased brain pH as a transdiagnostic endophenotype. However, the degree of brain pH reduction may differ among the disorders. We observed a Hedges’ *g* of −0.23 (*p* = 0.0056; Fig. 2) in this MDD study. Previously, we analyzed postmortem brain pH from nine datasets of schizophrenia and five from bipolar disorder, finding significant decreases in pH in both disorders using a two-way ANOVA (not a systematic review) [4]. Upon reanalyzing the same datasets to calculate Hedges’ *g*, we found values of −0.60 (*p* < 0.0001) for schizophrenia and −0.34 (*p* = 0.0040) for bipolar disorder. Although these Hedges’ *g* data cannot be directly compared due to the different conditions, the effect size of pH reduction in MDD may be relatively small, especially compared to schizophrenia. This idea is also suggested by transcriptomic analysis, where gene expression changes associated with increased acidity in the brain were more pronounced in schizophrenia and bipolar disorder than in MDD [10].

Our results indicated that brain pH was predominantly decreased in female patients with MDD (Fig. 3A). However, subgroup analyses revealed differences in the duration of illness and the proportion of subjects with recurrent episodes between female and male patients. Female patients have been known to experience more severe conditions compared to males, including a higher ratio of first-onset depressive episode, a higher ratio of recurrent and chronic depression [35], and more severe cognitive dysfunction [36]. Consistently, we observed a longer duration of illness and a trend toward a higher proportion of subjects with recurrent episodes in female patients compared to male patients in this study. Decreased brain pH was observed in patients with recurrent episodes, but not those with a single episode, and in patients with longer illness duration. Therefore, a higher proportion of subjects with recurrent episodes and a longer illness duration among female patients compared to male patients [35] may account for the observed female preponderance of decreased brain pH in MDD. Additionally, a higher proportion of subjects with a single episode, who showed brain pH levels comparable to controls, may partially explain the results showing no significant difference in pH between all patients and controls in male subjects (Fig. 3A).

As discussed above, we found lower brain pH levels in patients with recurrent episodes and those with a longer illness duration. This suggests significant effects of disease history they experienced before death on brain pH changes in MDD. Regarding the effect of disease state at the time of death on brain pH, we observed no significant differences in brain pH levels among patients in remitted, mild/moderate, or severe depressed states at the time of death. These findings suggest that decreased brain pH may be a trait of patients with MDD, influenced by the recurrence of episodes and illness duration, and persisting during remission, rather than a state at the time of death. In view of clinical manifestations, deficits in cognitive function, including working memory impairments, are hypothesized to worsen with repeated episodes of depression and persist during remission [37]. This pattern is similar to that of decreased brain pH suggested in patients with MDD. Our large-scale study using various animal models of neuropsychiatric disorders suggests that decreased brain pH is predominantly associated with working memory impairments [18]. These findings suggest that decreased brain pH may be a trait of patients with MDD associated with the working memory deficits observed in patients, although the causal relationship remains unclear.

Previous studies have shown that brain pH does not significantly vary across regions in non-psychiatric subjects [30, 38]; however, it is unclear whether there is region-specificity in the decrease of pH in the brains of patients with MDD. Our results suggested that brain pH is decreased in the cerebral cortices but not in the cerebellum. A previous meta-analysis of whole-brain resting-state functional magnetic resonance imaging (fMRI) studies has indicated increased brain activity in several regions of the cerebral cortex but a decrease in the cerebellum in patients with MDD compared to healthy controls, as assessed by the amplitude of low-frequency fluctuation (ALFF), which reflects cortical excitability [39]. Another meta-analysis of fMRI studies also suggested hyperactivation in the prefrontal cortical region (ventromedial PFC), but not in the cerebellum, in MDD [40]. Such region-specific increase in neural activity in the cerebral cortex may underlie the decrease in pH in these regions. Since a small number of datasets were analyzed in this study, future studies with lager sample size are required to confirm the regional difference in pH changes in MDD. Furthermore, it is necessary to investigate pH changes in more specific cortical areas, as well as in other brain regions implicated in the pathology of depression, such as the hippocampus and amygdala [41, 42].

In our meta-analysis, we observed high heterogeneity of brain pH values among datasets (Fig. 2), which may be due to differences in protocols for sampling and preservation of tissues and measurement of pH across various laboratories. However, pH values obtained through different measurement protocols employed within the same laboratory have been shown to be highly significantly correlated [32], suggesting a minimal effect of protocol variations on pH values. Even if pH values vary depending on the measurement protocol, the samples for patients and controls in each study should have been measured using the same protocol. Therefore, the results of our analysis, where standardized mean differences between patients and controls calculated within each dataset were meta-analyzed, are unlikely to be affected by differences in measurement protocols.

Researches on schizophrenia and bipolar disorder have suggested that increased lactate levels are closely related to decreased brain pH [6]. Brain lactate levels are thought to be increased by neuronal hyperactivity [43, 44] and metabolic abnormalities due to mitochondrial dysfunction [6]. Indeed, reports indicate increased neuronal activity [39, 40] and brain metabolic abnormalities [45, 46] in depressive disorder, which may increase lactate levels [25, 26] and hence decrease brain pH in the disorder. In our large-scale analysis of animal models of neuropsychiatric disorders, a strong negative correlation between pH and lactate levels was observed, with lactate potentially accounting for 33.2% (*r* = −0.58) of the pH decrease based on the regression coefficient in a linear regression model [18]. In a focused analysis of major depression models, this contribution was estimated at 47.9% (*r* = −0.69; Fig. S4C). In human postmortem brain studies, the correlation between brain pH and lactate levels found to be *r* = −0.53 [5], suggesting that lactate contributes 28.1% to pH changes. Postmortem brain pH is also suggested to be affected by tissue integrity, although this notion remains controversial, as a high pH does not necessarily guarantee intact tissue RNA [47]. We estimated the contribution ratio of tissue integrity, as assessed by RIN, on tissue pH changes based on correlation coefficients. The contribution ratios were 2.6% (*r* = 0.16; this study), 6.8% (*r* = 0.2617) [47], 12.2% (*r* = 0.349) [30], and 35.8% (*r* = 0.598) [31]. Additionally, the contribution of other factors, such as neuronal activity-regulated production of carbon dioxide, another metabolic acid, should be considered in decrease in brain pH [48, 49]. While lactate is an important regulator of brain pH [6], many other factors may exert additional effects on the decreased in brain pH.

In this meta-analysis, we excluded studies that reported that brain pH was matched between patient and control groups within each study. However, the studies that did, in fact, perform the matching process but did not mention it might be included in this analysis. If such studies exist, they could affect our meta-analysis results, potentially reducing the extent of the observed decrease in pH in patients with MDD compared to control subjects.

Regarding the potential risk of bias in this study, the Egger’s regression test of the asymmetrical funnel plot indicated no significant publication bias among all datasets used in this study. Furthermore, we relied on demographic data, but not the main outcomes of each study. Therefore, the typical publication bias and outcome bias are likely minimized in this study.

A significant limitation in this study, regarding potential confounding factors, is the lack of consideration of agonal state and medication use, as information about these factors was rarely available. Previous studies have reported that individuals who experience prolonged agonal states exhibit lower brain pH [29, 38, 50–52], while others did not show a significant effect of agonal state on brain pH [30, 53]. If agonal state affects brain pH and was enhanced in the patients with MDD compared to controls in the used datasets, we cannot rule out the possibility that decreased pH was due to the enhanced agonal states in MDD. Furthermore, we did not consider in this study the effects of medication as a potential confounding factor. Studies in animals have proposed that chronic antipsychotic treatment may decrease brain pH by increasing lactate levels [5], and a significant proportion of patients with schizophrenia or bipolar disorder receive long-term antipsychotic treatment. On the other hand, in human studies, no significant influence of medications on brain pH or lactate levels was observed among patients with schizophrenia and bipolar disorder [4, 5, 54]. Moreover, medication-free patients with bipolar disorder also showed a decreased brain pH [11] and increased lactate levels [55] compared to corresponding controls by MRS imaging. These human studies suggest that the use of antipsychotic medications is not a major factor in regulating brain pH and lactate levels in schizophrenia and bipolar disorder. However, little has been known about effects of antidepressants on brain pH, which should be addressed in the future study.

It is technically difficult to exclude the influence of potential confounding factors in human studies. Animal models used in our previous studies were drug-naïve, with equivalent agonal states, postmortem intervals, and ages within each strain and condition [4, 18]. Therefore, studies in animal models can help confirm whether changes in brain pH and lactate levels are involved in the pathophysiology of neuropsychiatric disorders. In those studies, we demonstrated decreased brain pH and increased lactate levels in various animal models of neuropsychiatric disorders, including those with face and construct validity for depressive disorder, such as SERT KO mice [19, 20], mice exposed to social defeat stress [21, 22], and mouse models of comorbid depression in diabetes mellitus and colitis [23, 24] (Fig. S4) [18]. However, not all the animal models of depression examined exhibited decreased brain pH. In all the animal models, except for SERT KO mice, which is mixed gender, male mice were analyzed (Fig. S4) [18]. Considering our results suggesting a female preponderance of decreased brain pH in patients with MDD, measuring brain pH in female mice of depression models would be of interest. While the sex differences remain to be addressed, findings in animal models support the idea that decreased brain pH and increased lactate levels are involved in the underlying pathophysiology of the disorder and are not mere artifacts.

In summary, there may be a decrease in brain pH in patients with MDD compared to healthy controls. However, the interpretation of these results could potentially be confounded by various pre- and postmortem factors. In this context, it is noteworthy that brain pH was decreased in several animal models of depressive disorder, which are free of potential confounding factors inherent in human studies. The findings of animal studies lend support to the idea that a decrease in brain pH is associated with the underlying pathophysiology of the disorder, rather than mere an artifact. Further studies are required to determine whether such metabolic alterations leading to decreased pH are associated with either beneficial or detrimental effects on psychiatric conditions.

## Supplementary information

Supplementary Figures

PRISMA checklist

Supplementary Tables
