# Supplementary material for: Postmortem evidence of decreased brain pH in major depressive disorder: a systematic review and meta-analysis
Source: Transl Psychiatry. 2024 Nov 4;14:460. doi: 10.1038/s41398-024-03173-7 (PMC11535390; doi:10.1038/s41398-024-03173-7)
Supplement: Supplementary file 1 — Supplementary Figures [file 41398_2024_3173_MOESM1_ESM.docx]

**Postmortem evidence of decreased brain pH in major depressive disorder: a systematic review and meta-analysis**

Hideo Hagihara^1*^ and Tsuyoshi Miyakawa^1^

^1^Division of Systems Medical Science, Center for Medical Science, Fujita Health University, Toyoake, Japan

**Supplementary Figures**

**Figure S1. Funnel plot of the included datasets.** Each plot represents a dataset. SE, standard error; SMD, standardized mean difference.

**Figure S2. Effects of available variables on brain pH levels. A–C** Effect of suicide as a manner of death on brain pH levels in patients with MDD. Scatter plot of z-score-transformed pH values of control subjects (CON), patients with MDD who died by suicide (MDD-S), and those who did not (MDD-NS) in the combined female and male datasets (A), female datasets (B), and male datasets (C). A total of 1102 samples from 20 datasets were analyzed. The z-scores were calculated within each dataset. **D–F** Scatter plot of z-score-transformed pH values of controls, patients with MDD in full or partial remission, those in mild or moderate condition, and those in severe condition in the combined female and male datasets (D), female datasets (E), and male datasets (F). A total of 165 samples from two datasets were analyzed. The z-scores were calculated within each dataset. **G** Correlation between brain pH and RNA integrity. Scatter plot showing the correlation between z-score-transformed pH values and RNA integrity number (RIN). The z-scores were calculated within each dataset. A total of 969 samples from 18 datasets were analyzed. Red line indicates the linear regression. *r*, Pearson’s correlation coefficient. **H** Scatter plot of RIN of patients with MDD and controls. A total of 929 samples from 18 datasets were analyzed. Red bars indicate mean values for each group (A–F, H). The p-values from Tukey’s multiple comparison test following one-way ANOVA (A, B), Pearson’s correlation coefficient (G), and two-way ANOVA (H) are shown. ^*^*p* < 0.05, ^**^*p* < 0.01.

**Figure S3. Effects of brain region and sex differences on tissue pH levels. A–C** Scatter plots of z-score-transformed pH values of cerebral cortical samples in the combined female and male datasets (A), female datasets (B), and male datasets (C). A total of 238 samples from five datasets were analyzed. **D–F** Scatter plots of z-score-transformed pH values of cerebellar samples in the combined female and male datasets (D), female datasets (E), and male datasets (F). A total of 206 samples from six datasets were analyzed. The z-scores were calculated within each dataset. Red bars indicate mean values for each group. The p-values from the unpaired t-test are shown. ^*^*p* < 0.05, ^**^*p* < 0.01.

**Figure S4. Decreased brain pH in animal models of depression.** **A, B** Bar graphs of pH (**A**) and lactate levels (**B**) in the brains of serotonin transporter knockout (SERT KO) mice, glucocorticoid receptor (Nr3c1)-overexpressing mice, mice exposed to repeated social defeated stress (SDS), mice treated with streptozotocin (STZ) and/or exposed to chronic restraint stress (RS), mice chronically treated with corticosterone (CORT), and mice treated with dextran sulfate sodium (DSS). ^*^*p* < 0.05, ^**^*p* < 0.01, unpaired t-test; ^#^*p* < 0.05, ^##^*p* < 0.01, two-way ANOVA (factors: genotype/condition and batch); NS, not significant. **C** Scatter plot showing correlations between pH and lactate levels in the mouse brain in *z*-score-based analysis. The data was adapted from Hagihara et al., 2024 [18].
